# RBPJ Is a Novel Target for Rhabdomyosarcoma Therapy

**DOI:** 10.1371/journal.pone.0039268

**Published:** 2012-07-09

**Authors:** Hiroko Nagao, Takao Setoguchi, Sho Kitamoto, Yasuhiro Ishidou, Satoshi Nagano, Masahiro Yokouchi, Masahiko Abematsu, Naoya Kawabata, Shingo Maeda, Suguru Yonezawa, Setsuro Komiya

**Affiliations:** 1 Department of Orthopaedic Surgery, Graduate School of Medical and Dental Sciences, Kagoshima University, Kagoshima, Japan; 2 The Near-Future Locomotor Organ Medicine Creation Course (Kusunoki Kai), Graduate School of Medical and Dental Sciences, Kagoshima University, Kagoshima, Japan; 3 Department of Human Pathology, Field of Oncology, Graduate School of Medical and Dental Sciences, Kagoshima University, Kagoshima, Japan; 4 Department of Medical Joint Materials, Graduate School of Medical and Dental Sciences, Kagoshima University, Kagoshima, Japan; Cedars-Sinai Medical Center, United States of America

## Abstract

The Notch pathway regulates a broad spectrum of cell fate decisions and differentiation processes during fetal and postnatal development. In addition, the Notch pathway plays an important role in controlling tumorigenesis. However, the role of *RBPJ*, a transcription factor in the Notch pathway, in the development of tumors is largely unknown. In this study, we focused on the role of *RBPJ* in the pathogenesis of rhabdomyosarcoma (RMS). Our data showed that Notch pathway genes were upregulated and activated in human RMS cell lines and patient samples. Inhibition of the Notch pathway by a γ-secretase inhibitor (GSI) decreased the *in vitro* proliferation of RMS cells. Knockdown of *RBPJ* expression by RNAi inhibited the anchorage-independent growth of RMS cells and the growth of xenografts *in vivo*. Additionally, overexpression of *RBPJ* promoted the anchorage-independent growth of RMS cells. Further, we revealed that *RBPJ* regulated the cell cycle in RMS xenograft tumors and decreased proliferation. Our findings suggest that *RBPJ* regulates the RMS growth, and that the inhibition of *RBPJ* may be an effective therapeutic approach for patients with RMS.

## Introduction

Rhabdomyosarcoma (RMS) is the most common soft tissue sarcoma in children and adolescents [1], [2], [3]. Pediatric RMS can be divided into 2 major subtypes, embryonal RMS (eRMS) and alveolar RMA (aRMS). The cure rates for patients with nonmetastatic RMS have improved significantly from an estimated 25% in 1970 to 75% at present. Prognosis for RMS is dependent on the anatomic site of the primary tumor, age, completeness of resection, presence and the number of metastatic sites, and histological and biological characteristics of the tumor cells [4], [5]. The advances in the understanding of tumor biology may lead to the development of novel clinically relevant therapeutic targets in the near future.

The Notch signaling cascade is highly conserved and plays a crucial role in the self-renewal of stem cells, cell fate determination, epithelial cell polarity, adhesion, cell division, and apoptosis [6], [7], [8]. The mammalian family of Notch receptors consists of 4 members (*NOTCH1-4*) and the ligand family consists of 5 members (*JAGGED 1/2* and *DELTA 1/3/4*). In the absence of ligand binding, the Notch receptors are inactive. Upon ligand binding, the Notch receptor is cleaved in 2 sequential steps. The cleavage events release the intracellular domain of the Notch receptor (NICD), and the NICD regulates the downstream target genes via the DNA-binding factor, *RBPJ/CBF1* [9], [10]. The transcriptional regulator *RBPJ* is a highly conserved DNA-binding protein that plays a central role in canonical Notch signaling [11].

Recently, alterations in the Notch pathway have been observed in different solid tumors, including breast cancer, ovarian cancer, melanoma, glioblastoma, and lung and pancreatic cancer [12], [13], [14]. In addition, aberrant activation of the Notch-RBPJ pathway is involved in Epstein–Barr virus (EBV) infection [15], [16], T-lymphoblastic leukemia (T-LL), and gliomas [17], [18].

We previously reported that inhibition of the Notch pathway suppressed the growth of osteosarcoma by regulation of cell cycle [19]. In this study, we found that the Notch pathway was also functionally activated in human RMS, and a γ-secretase inhibitor (GSI) X reduced the *in vitro* proliferation of RMS cells. Moreover, we show that inhibition of *RBPJ* expression prevents the growth of RMS *in vitro* and *in vivo*.

## Materials and Methods

### Cell Lines

RD and KYM-1 cell lines were obtained from the Health Science Research Resources Bank (HSRRB, Osaka, Japan). RMS-YM cell line was obtained from Riken Bioresource Center (Tsukuba, Japan). HSKMc cell line was purchased from TOYOBO (Osaka, Japan). RD and KYM-1 cell lines were cultured in Dulbecco’s modified Eagle’s medium (DMEM) supplemented with 10% fetal bovine serum (FBS), 100 U/mL penicillin, and 100 µg/mL streptomycin. RMS-YM cell line was cultured in RPMI 1640 medium supplemented with 10% FBS, 100 µM nonessential amino acids (NEAA), 20 mM HEPES, 100 U/mL penicillin, and 100 µg/mL streptomycin. HSKMc cell line was cultured in skeletal muscle cell growth medium (TOYOBO, Osaka, Japan). All cells were maintained at 37°C in 5% CO_2_.

### Patient Specimens

Human eRMS biopsy specimens were collected from primary lesions before any diagnostic or therapeutic treatment. Human skeletal muscle tissues were collected from patients undergoing operation for scoliosis. The study protocol was approved by the institutional Review Board of Kagoshima University. Informed consent was obtained from all patients.

### Real-time PCR Analysis

Real-time PCR analysis was performed as previously described [20]. Total RNA was extracted from cell lines and tissue specimens using miR-Vana RNA isolation kit or TRIzol (Invitrogen, CA, USA) and was reverse transcribed using ReverTra Ace -α- (TOYOBO, Osaka, Japan). cDNA was amplified by real-time PCR using SYBR Green (Life Technologies, NY, USA). Real-time PCR was performed on MiniOpticon™ (Bio-Rad, Tokyo, Japan). The comparative Ct (ΔΔCt) analysis was performed to evaluate the fold change of mRNA expression, using the expression of *ACTB* as a reference. All PCR reactions were performed in triplicate. All primers were designed. using Primer 3 software. The following primers were used: *ACTB*, 5′-AGAAAATCTGGCACCACACC-3′ and 5-AGAGGCGTACAGGGATAGCA-3′; *NOTCH1*, 5′-GTGACTGCTCCCTCAACTTCAAT-3′ and 5′-CTGTCACAGTGGCCGTCACT-3′; *NOTCH2*, 5′-GTGTCAGAATGGAGGGGTTTG-3′ and 5′-ATTGCGGTTGGCACAGG-3′; *NOTCH3*, 5′-CAACCCGGTGTACGAGAAGT-3′ and 5′-GAACGCAGTAGCTCCTCTGG-3′; *NOTCH4*, 5′-CCATTGACACCCAGCTTCTT-3′ and 5-GCTGAACAGAAGTCCCGAAG-3′; *JAG1*, 5′-CAGATTTCCTTGTTCCCTTGCT-3′ and 5′-CGTTGTTGGTGGTGTTGTCC-3′; *DLL1*, 5′-CCTACTGCACAGAGCCGATCT-3′ and 5′-GCAGGTGGCTCCATTCTTGC-3′; *HES1*, 5′-AGGCGGACATTCTGGAAATG-3′ and 5′-CGGTACTTCCCCAGCACACTT-3′; *HEY1*, 5′-CGAGGTGGAGAAGGAGAGTG-3′ and 5′-CTGGGTACCAGCCTTCTCAG-3′; *RBPJ*, 5′-CGCATTATTGGATGCAGATG-3′ and 5′-CAGGAAGCGCCATCATTTAT-3′; *Cyclin D*, 5′-CAGAAGTGCGAGGAGGAGGT-3′, and 5′-CGGATGGAGTTGTCGGTGT-3′; *Cyclin E*, 5′-CCACACCTGACAAAGAAGATGATGAC-3′ and 5′-GAGCCTCTGGATGGTGCAATAAT-3′; *E2F1*, 5′-ATGTTTTCCTGTGCCCTGAG-3′ and 5′-ATCTGTGGTGAGGGATGAGG-3′; *SKP2*, 5′-TGGGAATCTTTTCCTGTCTG-3′ and 5′-GAACACTGAGACAGTATGCC-3′; *p21*, 5′-GACACCACTGGAGGGTGACT-3′ and 5′-ACAGGTCCACATGGTCTTCC-3′.

### Cell Proliferation Assay

Cell proliferation assay was performed as previously described [21]. We seeded 1×10^3^ cells (RD) or 3×10^3^ cells (KYM-1)/100 µL in a 96 -well plate. Next day, the cells were placed in fresh medium containing the indicated concentration of the GSI X (CALBIOCHEM, Basel, Switzerland), GSI XX (CALBIOCHEM, Basel, Switzerland) or DMSO and were cultured for 3–4 days. Cell growth were measured daily by performing WST-1 assay (Roche, Basel, Switzerland).

### Plasmid Constructs and Gene Transfer

Control siRNA (S20C-0600) was purchased from B-Bridge International (Cupertino, USA) and RBPJ siRNA (sc-38214) was purchased from Santa Cruz Biotechnology (CA, USA). All siRNA transfection experiments were performed using Lipofectamine RNAiMAX (Invitrogen, CA, USA) transfection reagent according to the manufacturer’s protocol. Control or RBPJ shRNA (KH06319P) were purchased from SuperArray Biosciences (MD, USA). pCMV6-Entry Vector (PS100001) and RBPJ expression vector (RC204791) were purchased from Origene (Maryland, USA). All plasmid transfection experiments were performed using FuGENE6 (Roche, Basal, Switzerland) transfection reagent according to the manufacturer’s protocol. Tranfected cells were selected in 700 µg/mL neomycin or 0.4 ng/µL puromycin. Stable cell lines were then used for colony formation assay and in vivo experiments.

### Colony Formation Assay

Colony formation assay was performed as previously described [22]. Cells were suspended in DMEM containing 0.33% soft agar and 5% FBS and then were plated on a 0.5% soft agar layer. Cells were cultured at a density of 2×10^4^ cells per well in 6-well plates. After 2–3 weeks (RBPJ siRNA/RD: 2 weeks, RBPJ/RD: 3 weeks), the number of colonies was counted. Every experiment was performed in triplicate, and all experiments were performed 3 times.

### Western Blotting Analysis

Western blotting analysis was performed as previously described [23].

Cells were lysed using NP40 buffer, including 0.5% NP40, 10 mM Tris-HCl (pH 7.4), 150 mM NaCl, 3 mM pAPMSF (Wako Chemicals, Kanagawa, Japan), 5 mg/mL aprotinin (Sigma, StLouis, USA), 2 mM sodium orthovanadate (Wako Chemicals, Kanagawa, Japan), and 5 mM EDTA. Lysates were boiled with sodium dodecyl sulfate (SDS) sample buffer, separated by SDS-polyacrylamide gel electrophoresis (PAGE) (Bio-Rad, Tokyo, Japan), and transferred to a polyvinylidene fluoride (PVDF) membrane (Caliper LifeSciences, CA, USA). The membranes were blocked in 5% nonfat dry milk TBST buffer and incubated in primary antibodies diluted in TBST for 1 h at room temperature or overnight at 4°C. Blots were washed using TBST buffer and incubated with horseradish peroxidase-conjugated secondary antibodies (Cell Signaling Technology) in TBST buffer for 45 min at room temperature. Immunocomplexes were visualized using an enhanced chemiluminescence kit (GE Healthcare, Tokyo, Japan). Primary antibodies were RBPJ (1∶300, ab33065, abcam), PARP (1∶1000, #9542, Cell Signaling) and α-tubulin (1∶1000, DM1A, Sigma-Aldrich).

### Animal Studies

Xenograft experiments were performed as previously described [24]. Briefly, control or RBPJ shRNA-transfected RD cells (1×10^6^) were suspended in 100 µL Matrigel (BD, NJ, USA). Cell suspensions were subcutaneously inoculated in 5-week-old nude mice (Japan SLC, Inc). Tumor size was calculated weekly using the formula LW^2^/2 (with L and W representing the length and width of tumors). Kaplan–Meier analysis was performed using Kaplan 97 software. All animal experiments were performed in compliance with the guidelines and approved by the Animal Science Laboratory, Frontier Science Research Center,Kagoshima University.

### Immunohistochemistry

For Ki-67 staining, antigen retrieval was performed using CC1 antigen retrieval buffer (Ventana Medical Systems, Tucson, AZ, U.S.A.) for all sections. Following incubation with the primary antibody against Ki-67 (MIB-1, DAKO, dilution rate at 1∶50), sections were stained on the Ventana automated slide stainer (Benchmark XT) using the Ventana diaminobenzidine detection kit (Ventana Medical Systems). Ki67 immunostainings were scored by counting at least 1000 cells in 5 randomized fields. Every stained nucleus was considered positive, irrespective of intensity.

For detection of apoptotic small bodies, cells were fixed by 4% paraformaldehyde for 20 min, washed in PBST (PBS containing 0.05% Tween20), and then permeabilized in PBS containing 0.2% Triton X-100 for 10 min. After the wash, the cells were treated with PBS containing 10 µg/mL Hoechst 33342 dye (Molecular Probes, Oregon, U.S.A) for 30 min, and then were washed. The apoptotic cells were visualized by fluorescence microscopy (Leica Microsystems, Wild Heerbrugg, Switzerland).

### Statistical Analysis

All the data are expressed as mean ± SD. Statistical analysis was performed using the Student’s *t* test using Microsoft Office Excel or Kaplan 97. P<0.05 was considered significant.

## Results

### Notch Pathway Genes are Upregulated in Tissue Specimens of Patients with Rhabdomyosarcoma

**Figure 1:**
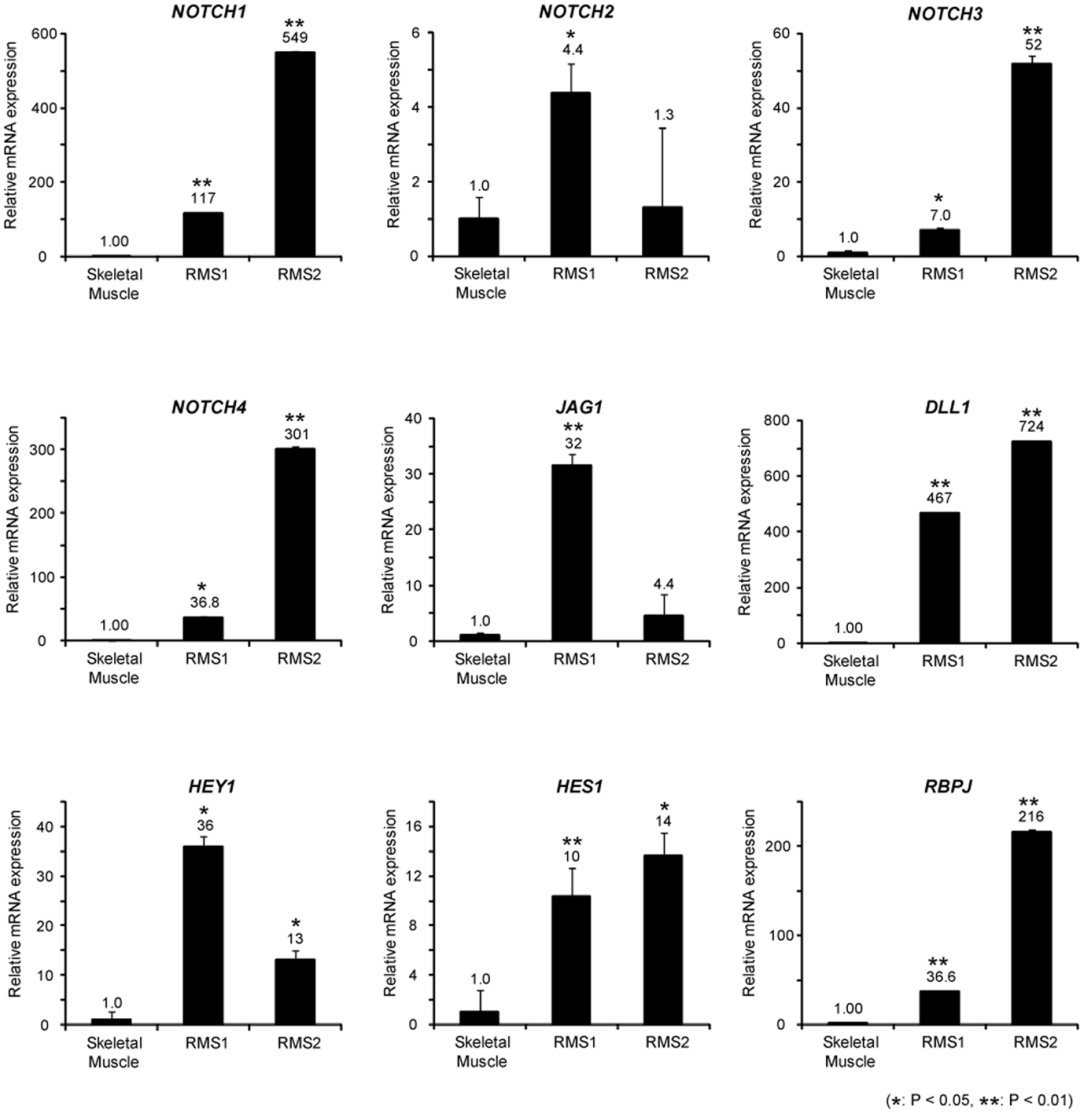
Notch pathway molecules are overexpressed in rhabdomyosarcoma cells. Notch pathway genes (receptors *NOTCH1-4*, ligands *JAG1* and *DLL1*, target genes *HES1* and *HEY1*, and transcription factor *RBPJ*) were assessed by real-time PCR in a normal human skeletal muscle specimen and 2 human RMS biopsy specimens. The Ct values of all RMS samples were normalized to those of *ACTB*. The values of the human RMS specimens were compared with those of the human skeletal muscle sample, which is defined as a relative expression of 1.0. Columns, mean values of 3 independent experiments; bar, SD. *p<0.05, **p<0.01.

We assessed the status of the Notch pathway in RMS by determining the expression of genes in the Notch pathway; we performed real-time PCR to determine the expression of these genes in normal human skeletal muscle specimen and 2 human eRMS specimens. RMS1 and RMS2 showed strong expression of Notch receptors *NOTCH1*, *NOTCH3*, and *NOTCH4* in RMS specimens. Additionally, Notch ligands *JAG1* and *DLL1*, target genes *HEY1* and *HES1*, and transcription factor *RBPJ* were significantly upregulated in RMS (Fig. 1). Further, we showed that Notch pathway molecules are upregulated in RMS cell lines (Fig. S1). These findings suggest that the Notch pathway is activated in human RMS.

### Downregulation of the Notch Pathway by GSI X Suppresses Rhabdomyosarcoma Cell Proliferation

**Figure 2:**
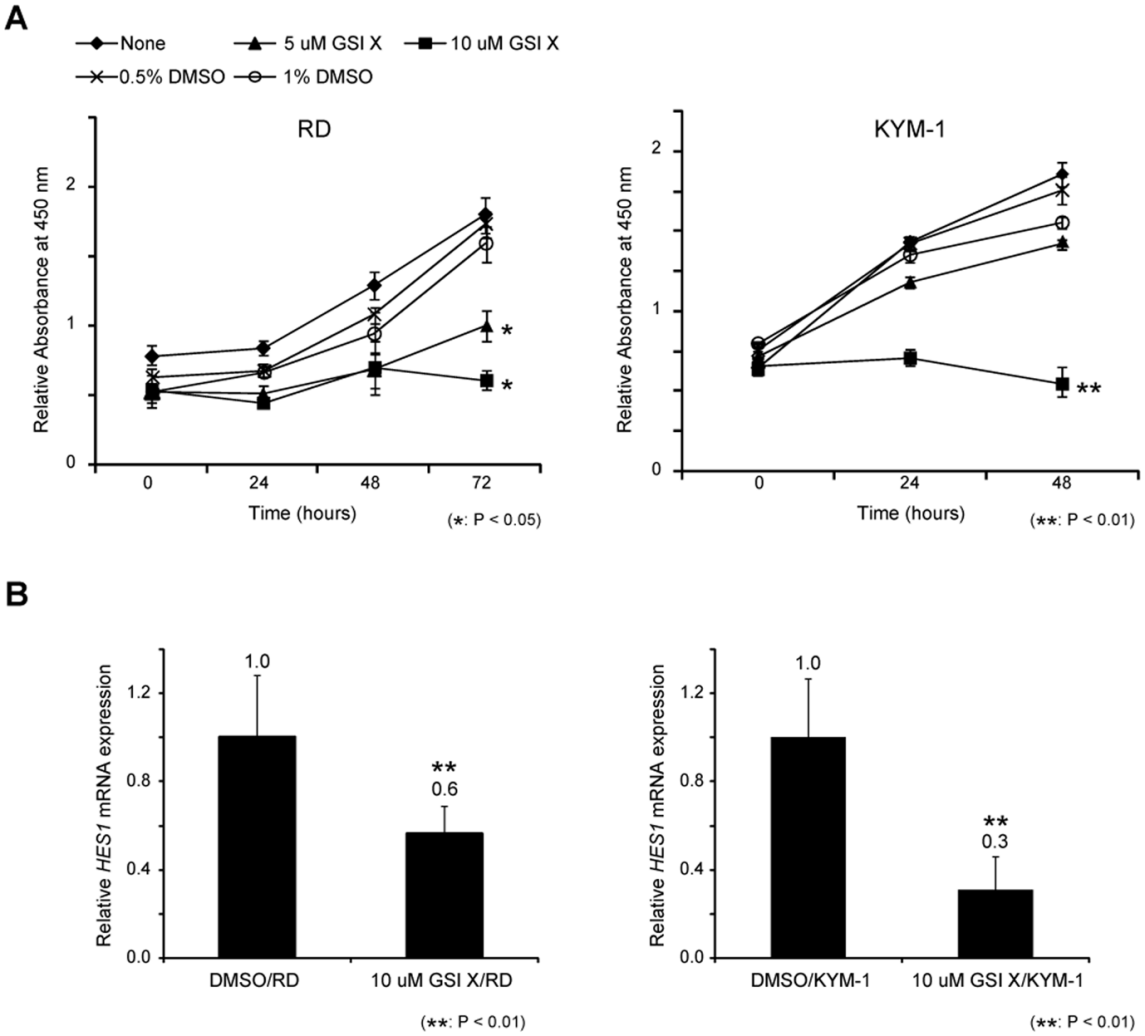
Effects of GSI X on the proliferation of rhabdomyosarcoma cells. **A**, Proliferation of RD and KYM-1 cells treated with GSI X or equal volume of DMSO vehicle were measured by WST-1 assay. **B**, Expression of *HES1* mRNA was assessed by real-time PCR in RD cells (left) and KYM-1 cells (right) treated with 10 µM of GSI X for 24 hours. Columns and lines, mean values of 3 independent experiments; bar, SD. *p<0.05, **p<0.01.

To examine whether the Notch pathway contributes to RMS pathogenesis, we used GSI X and GSI XX which are potent inhibitor of Notch pathway. WST-1 assay revealed that the proliferation of RD and KYM-1 cells was inhibited by 10 µM GSI X (Fig. 2A). In addition, GSI XX prevented RD and KYM-1 proliferation (Fig. S2). We evaluated cell death by GSI X treatment. GSI X treatment did not promote the expression of cleaved PARP and formation of the apoptotic small bodies (Fig. S3). Furthermore, the Notch target gene *HES1* mRNA was downregulated by 10 µM GSI X, in RD and KYM-1 cell lines (Fig. 2B). These findings suggest that Notch pathway inhibition by GSI X treatment prevents the proliferation of RMS cells *in vitro*.

### *RBPJ* is Essential for the Growth of Rhabdomyosarcoma

**Figure 3:**
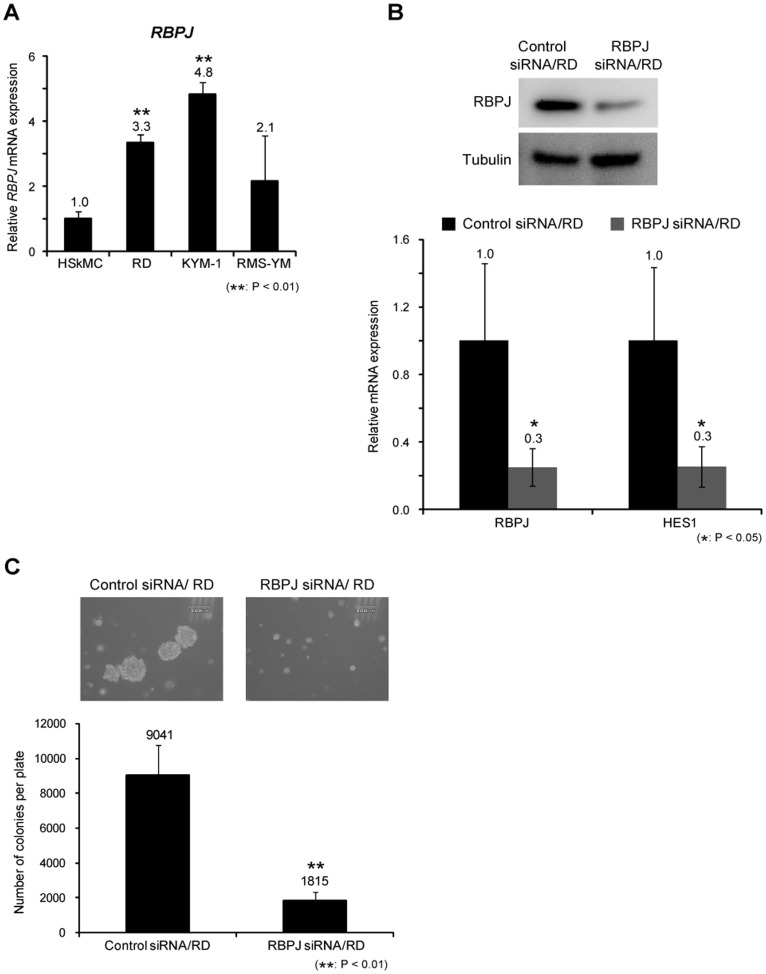
Knockdown of *RBPJ* suppresses anchorage-independent growth of rhabdomyosarcoma cells. A, The expression of *RBPJ* mRNA in RD cells was assessed by real-time PCR. The Ct values of all RMS cell lines were normalized to those of *ACTB*. The values of the RMS cell lines were compared with HSkMc cell, which is defined as a relative expression of 1.0. **B,** RBPJ protein levels in RD cells transfected with control and RBPJ siRNA were examined by western blotting analysis (top). *RBPJ* and *HES1* mRNA in RD cells transfected with control and *RBPJ* siRNA were assessed by real-time PCR analysis. Ct values of *RBPJ* and *HES1* were normalized to *ACTB*. The values of the cells transfected with *RBPJ* siRNA were compared to those the RD cells transfected with control siRNA, which is defined as a relative expression of 1.0 (bottom). **C,** Anchorage-independent growth of RD cells transfected with control and *RBPJ* siRNA were evaluated by colony formation assay. After 3 weeks, each of the colonies were counted and photographed. Scale bar is 200 µM. Columns, mean values of 3 independent experiments; bar, SD. *p<0.05, **p<0.01.

**Figure 4:**
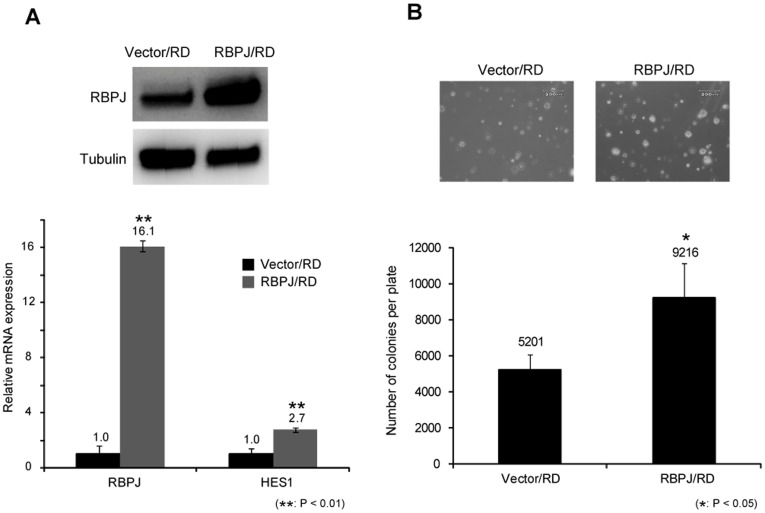
Overexpression of *RBPJ* promotes rhabdomyosarcoma cell growth. A, RBPJ protein levels in RD cells transfected with control vector and *RBPJ* overexpression vector were measured by Western blotting analysis (top). *RBPJ* and *HES1* mRNA in RD cells transfected with control vector and *RBPJ* overexpression vector were assessed by real-time PCR analysis. Ct values of *RBPJ* and *HES1* were normalized to *ACTB*. Comparison was made to the RD cells transfected control vector, which is defined as a relative expression of 1.0 (bottom). **B,** Anchorage-independent growth in RD cells transfected with control vector and *RBPJ* overexpression vector were evaluated by colony formation assay. Fourteen days later, the each colonies were counted and pictured. Scale bar is 200 µM. Columns, mean values of 3 independent experiments; bar, SD. *p<0.05, **p<0.01.

GSIs inhibit not only the Notch pathway but also other pathways [26], [27], [28]. We examined the function of the Notch pathway in RMS cell proliferation by analyzing the function of *RBPJ*. Real-time PCR revealed that *RBPJ* was upregulated 2.1 to 4.8-fold in RMS cell lines (Fig. 3A). To evaluate the function of *RBPJ* in RMS, we knocked down *RBPJ* expression by using siRNA. Efficacy of RNAi was confirmed by real-time PCR and western blotting assay, which showed that *RBPJ* RNAi decreased the expression of *RBPJ* mRNA and protein levels (Fig. 3B). Furthermore, knockdown of *RBPJ* decreased the expression of Notch target gene *HES1* mRNA in RD cells (Fig. 3B). RD cells transfected with *RBPJ* siRNA showed a significantly lower number of colonies in soft agar than those with control siRNA (Fig. 3C). In addition to loss-of-function of *RBPJ*, we examined the effects of forced expression of *RBPJ* in RMS cells. Forced expression of RBPJ increased the expression of downstream target gene *HES1* (Fig. 4A). The colony formation assay showed that forced expression of *RBPJ* led to formation of a greater number of colonies in soft agar than those with control vector (Fig. 4B). These findings show that *RBPJ* promotes the growth of human RMS cells *in vitro*.

**Figure 5:**
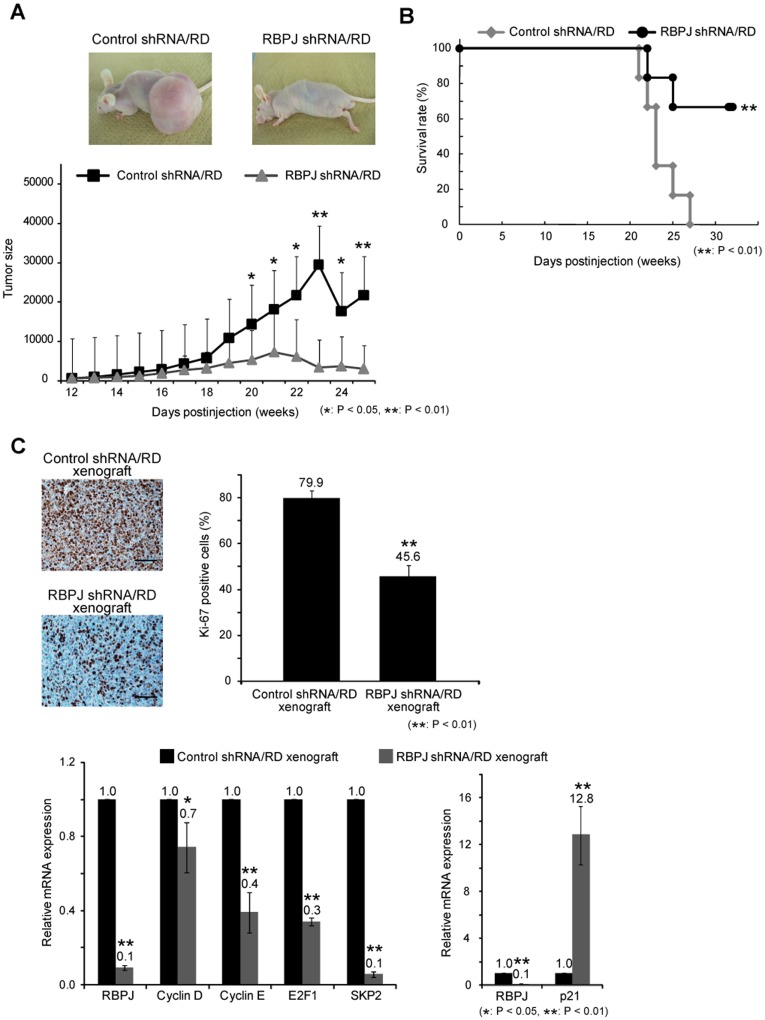
Knockdown of *RBPJ* inhibits the growth of rhabdomyosarcoma in nude mice. A, After transfection of control shRNA or *RBPJ* shRNA, 1×10^6^ RD cells were subcutaneously inoculated in nude mice (n = 7). Tumor size was calculated weekly by using the formula LW^2^/2 (with L and W representing the length and width of tumors). **B**, Survival rate of the mice injected with control shRNA- or *RBPJ* shRNA-transfected RD cells was assessed by Kaplan–Meier analysis. **C**, The number of Ki67-positive cells in control shRNA- or RBPJ shRNA-transfected xenograft were accessed by immnohistochemistry. Scale bar is 100 µm. Expression of Cell cycle-related genes (*CyclinD*, *CyclinE*, *E2F1*, *SKP2*, *p21*) were assessed by real-time PCR in control shRNA- or RBPJ shRNA-transfected xenograft. The Ct values of xenograft samples were normalized to those of *ACTB*. The values of the RBPJ shRNA-transfected xenograft was compared with those of the control shRNA sample, which is defined as a relative expression of 1.0. Columns, mean values of 3 independent experiments. Bar, SD. *p<0.05, **p<0.01.

### Knockdown of *RBPJ* Prevents the Growth of Rhabdomyosarcoma *in vivo*

We next investigated whether knockdown of *RBPJ* affects the growth of RMS cells *in vivo*. Knockdown of *RBPJ* by transfection of *RBPJ* shRNA significantly inhibited the growth of RD xenograft in nude mice as compared to that of control shRNA-transfected xenograft (Fig. 5A). Kaplan–Meier analysis revealed that knockdown of *RBPJ* showed a statistically significant improvement in the survival of mice (Fig. 5B). To examine whether *RBPJ* knockdown reduced cell proliferation *in vivo*, we examined the expression of Ki67 and cell cycle-related genes. The number of Ki67-positive cells in *RBPJ* shRNA-transfected xenograft was significantly lower than in control shRNA-transfected xenograft (Fig. 5C). Additionally, real-time PCR showed that the expression of the cell cycle accelerators, such as *Cyclin D*, *Cyclin E*, *E2F1*, and *SKP2* was decreased in *RBPJ* shRNA-transfected xenograft. In contrast, the expression of *p21*, a negative regulator of cell cycle, was increased in *RBPJ* shRNA-transfected xenograft (Fig. 5C). These findings suggest that *RBPJ* plays a critical role in the growth of RMS by regulation of the cell cycle *in vivo*.

## Discussion

The Notch pathway is involved in several cellular processes such as proliferation, differentiation, apoptosis, cell fate decision, and maintenance of stem cells [8], [12], [29]. The Notch pathway plays a role in many cancers [19], [30], [31], [32].

Our findings revealed that Notch pathway molecules were upregulated in clinical samples of eRMS were consistent with those reported in previous studies. Kuroda, K. *et al*. showed that activation of the *NOTCH1*-*RBPJ* pathway via the *DLL1* ligand was important for myogenic differentiation [33], [34]. We also found that the mRNA expression of *NOTCH1*, *DLL1*, and *RBPJ* is higher in eRMS specimens than in normal skeletal muscle specimens. Thus, the pathogenesis of RMS might be associated with the dysregulated activation of myogenic differentiation.

Recently, the Notch pathway has been reported to be highly active in human RMS [35], [36]. Roma J *et al*. reported that inhibition of the Notch pathway by GSIs reduced the invasiveness and metastasis of RMS *in vitro* [35]. In addition, Belyea *et al*. reported that Notch pathway inhibition by GSI and RNAi of *NOTCH1* or *HEY1* blocked RMS tumorigenesis [36]. GSIs, which were originally used in Alzheimer’s disease [37], are currently undergoing clinical trials for the treatment of several tumors [38], [39]. However, previous studies have shown that GSIs can kill breast cancer cells because of their nonspecific effect through their ability to inhibit the proteasome rather than blocking γ-secretase activity [25]. Additionally, GSIs proteolyze not only Notch receptor but also around 51 membrane proteins, including E-cadherin, VEGFR, and CXCL16 [40]. Furthermore, *HEY1* is involved in TGF-β pathway [41]. Thus, therapeutic strategies including treatment with GSIs or those targeting *HEY1* may affect other pathways. On the other hand, *RBPJ* acts only downstream of the Notch pathway, and nothing is known about its function in other pathways. Thus, we focused on the role of RBPJ to examine the bona fide function of the Notch pathway transcription factor in RMS tumorigenesis. The loss-of-function and gain-of-function of *RBPJ* indicated that *RBPJ* controled RMS cell growth *in vitro*. Although GSI treatment decreased the proliferation of RMS cells, knockdown of RBPJ did not decrease the proliferation in normal culture condition (data not shown). These findings suggest that GSI inhibit Notch pathway more intensively than RBPJ knockdown or might affect not only Notch pathway but also other signaling pathways. On the other hand, knockdown of RBPJ prevented RMS proliferation in soft agar (3D culture) and *in vivo*. In addition, knockdown of *RBPJ* caused significantly improved the survival of mice. These findings suggest that transcription of RBPJ activated by Notch pathway is essential for RMS proliferation in physiological conditions. Although these three studies inhibit Notch signaling by inhibitor or knockdown of different genes, all studies provide independent support for the idea that Notch pathway plays essential roles in RMS progression. Furthermore, Notch pathway plays essential roles in many cancers [19], [30], [31], [32]. Our results show that direct inhibition of *RBPJ* may offer a novel approach for inhibition of the Notch pathway not only in RMS but also in many cancers.

We showed that knockdown of *RBPJ* suppressed the expression of *Cyclin D*, *Cyclin E*, *E2F1*, and *SKP2*, whereas the expression of *p21* increased in *RBPJ* shRNA-transfected xenograft. SKP2, a subunit of the ubiquitin-ligase complex SCF^SKP2^, is necessary for the degradation of p21 at the G1/S transition and during S phase in the cell cycle [42]. p21 inhibits CDK4-Cyclin D and thus suppresses the phosphorylation of RB and the sequestration of E2F1 and Cyclin E [43]. Therefore, our findings suggest that the inhibition of RBPJ prevents RMS growth *in vivo* by regulation of G1/S transition of the cell cycle.

Several studies have shown the relation between the Notch pathway and tumor-initiating cells (TICs) [44], and Sullivan *et al*. reported that aldehyde dehydrogenase (ALDH) activity selected for lung TICs is dependent on the Notch pathway [45]. Thus, we confirmed ALDH activity for the Notch pathway in RMS cell lines. However, no significant difference was observed in ALDH-positive population between *RBPJ* shRNA- and control shRNA-transfected RD cells (data not shown). Previously, we reported that the RMS cell lines included fibroblast growth factor receptor 3 (FGFR3)-positive TICs, which have high tumorigenic potential *in vivo* [46]. Hence, we examined the expression of FGFR3 in *RBPJ* shRNA- or control shRNA-transfected RD; however, no significant difference was observed in FGFR3-positive population in these cell lines (data not shown). These findings suggest that *RBPJ* does not have the roles in maintenance of ALDH or FGFR3-positive TICs in RMS cell lines. Recently, it has been reported that RMS cells contains a CD133-positive TICs [47], [48]. Thus, further studies to explore that the relation between the Notch pathway and CD133-positive RMS TICs are needed to elucidate the pathogenesis of RMS.

Major advances have been made for understanding the interactions between the Notch pathway and other pathways during carcinogenesis [6], [7]. Schreck *et al*. reported that the Notch target *HES1* directly modulated *GLI1*, transcription factor of the Hedgehog pathway, in glioblastoma cells [49]. Additionally, we showed that the Hedgehog pathway was activated in human RMS [23]. Targeting both the Notch pathway and the Hedgehog pathway simultaneously may be more effective in eliminating RMS cells.

In conclusion, we revealed that the Notch pathway is functionally activated in RMS. Our findings show that inhibition of *RBPJ* prevents the growth of RMS *in vitro* and *in vivo*. These novel findings improve the understanding of the pathogenesis of RMS and suggest that *RBPJ* may be an attractive therapeutic target for patients with RMS.

## Supporting Information

Figure S1 **Notch pathway molecules are overexpressed in rhabdomyosarcoma cell lines.** Expression of Notch pathway genes (receptors *NOTCH1-4*, ligands *JAG1* and *DLL1*, target genes *HES1*, and *HEY1*) were assessed by real-time PCR in a human skeletal muscle cell line (HSkMC) and 3 human RMS cell lines. The Ct values of all RMS samples were normalized to those of *ACTB*. The values of the human RMS specimens were compared with those of the human skeletal muscle sample, which is defined as a relative expression of 1.0. Columns, mean values of 3 independent experiments. Bar, SD. *p<0.05, **p<0.01.
(TIF)

Figure S2 **GSI XX prevents proliferation of rhabdomyosarcoma cells.** RD and KYM-1 cells were treated with GSI XX or equal volume of DMSO vehicle. GSI XX treatment prevented the RMS proliferation.
(TIF)

Figure S3 **GSI X treatment did not promote rhabdomyosarcoma cell apoptotic cell death. A,** PARP and cleaved PARP protein levels in RD and KYM-1 cells were examine following 10 µM GSI X or equal volume of DMSO vehicle treatment. We used the PARP antibody which detect both full length PARP and cleaved PARP. Western blotting analysis revealed that GSI X treatment did not increase the expression of cleaved PARP. **B,** RD and KYM-1 cells were stained with Hoechst 33342 dye following 10 µM GSI X or equal volume of DMSO vehicle treatment. Apoptotic small body was not increased by GSI X treatment. Scale bar is 100 µm.
(TIF)
