# Supplementary figures and images for: RBPJ Is a Novel Target for Rhabdomyosarcoma Therapy
Source: PLoS One. 2012 Jul 9;7(7):e39268. doi: 10.1371/journal.pone.0039268 (PMC3392254; doi:10.1371/journal.pone.0039268)

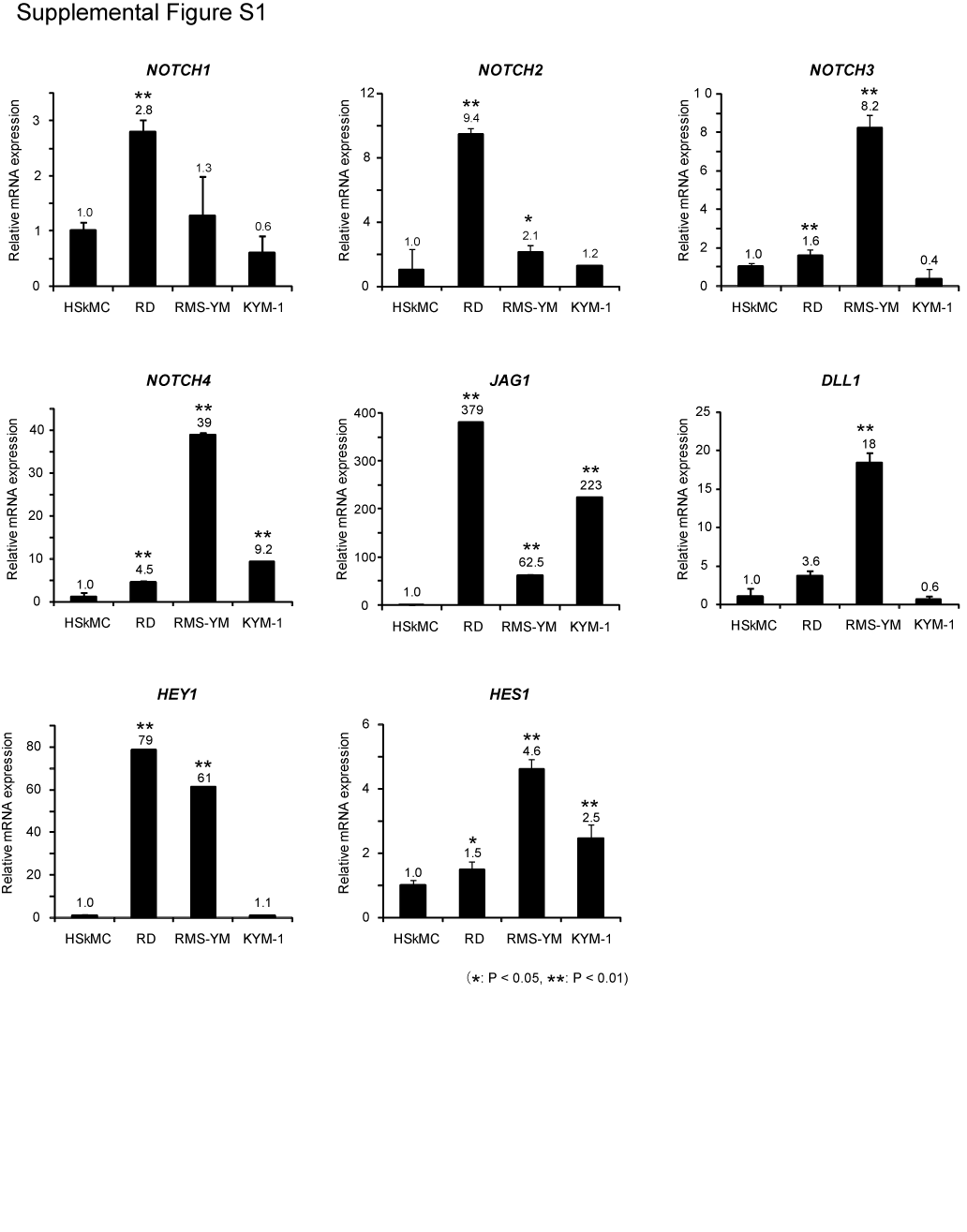

Supplement: Figure S1 — Notch pathway molecules are overexpressed in rhabdomyosarcoma cell lines. Expression of Notch pathway genes (receptors NOTCH1-4, ligands JAG1 and DLL1, target genes HES1, and HEY1) were assessed by real-time PCR in a human skeletal muscle cell line (HSkMC) and 3 human RMS cell lines. The Ct values of all RMS samples were normalized to those of ACTB. The values of the human RMS specimens were compared with those of the human skeletal muscle sample, which is defined as a relative expression of 1.0. Columns, mean values of 3 independent experiments. Bar, SD. *p<0.05, **p<0.01. (TIF) [file pone.0039268.s001.tif]

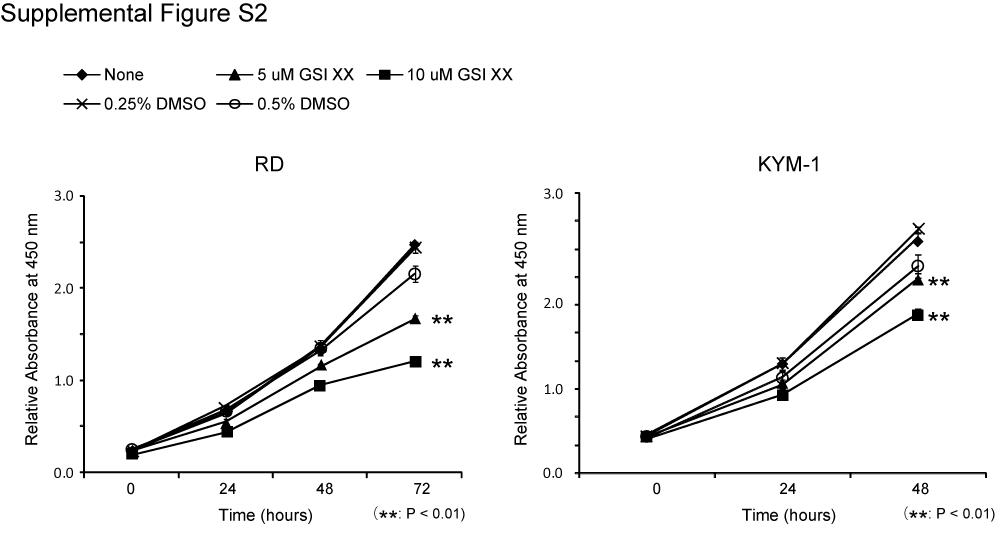

Supplement: Figure S2 — GSI XX prevents proliferation of rhabdomyosarcoma cells. RD and KYM-1 cells were treated with GSI XX or equal volume of DMSO vehicle. GSI XX treatment prevented the RMS proliferation. (TIF) [file pone.0039268.s002.tif]

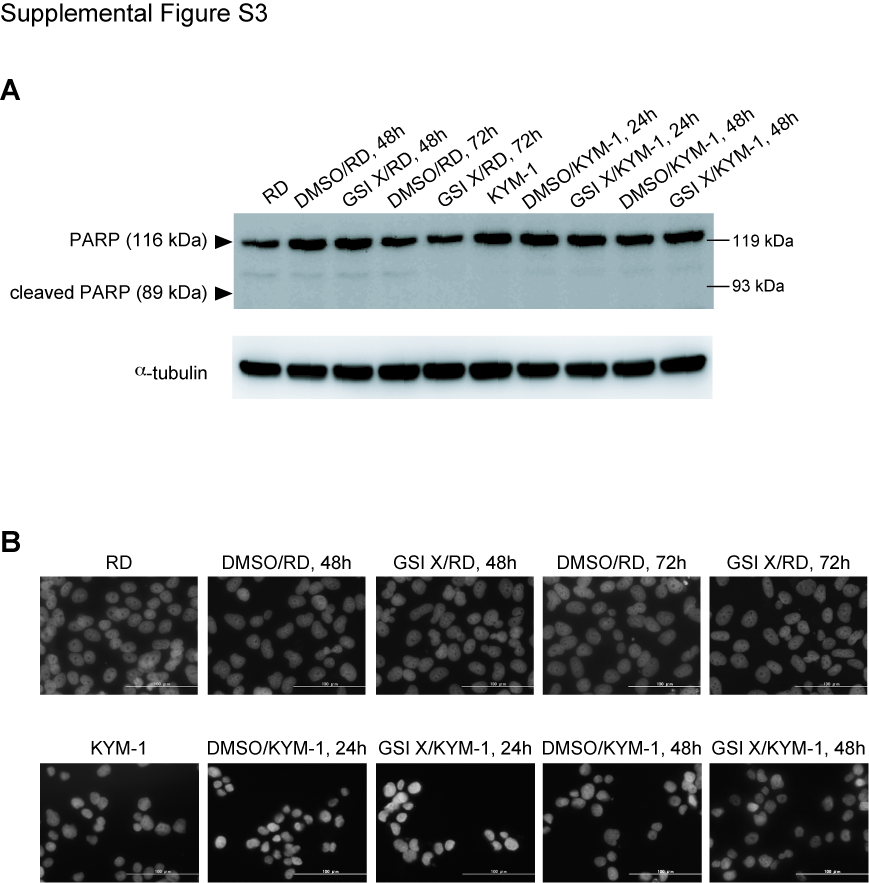

Supplement: Figure S3 — GSI X treatment did not promote rhabdomyosarcoma cell apoptotic cell death. A, PARP and cleaved PARP protein levels in RD and KYM-1 cells were examine following 10 µM GSI X or equal volume of DMSO vehicle treatment. We used the PARP antibody which detect both full length PARP and cleaved PARP. Western blotting analysis revealed that GSI X treatment did not increase the expression of cleaved PARP. B, RD and KYM-1 cells were stained with Hoechst 33342 dye following 10 µM GSI X or equal volume of DMSO vehicle treatment. Apoptotic small body was not increased by GSI X treatment. Scale bar is 100 µm. (TIF) [file pone.0039268.s003.tif]
